# Environmental Feedbacks and Engineered Nanoparticles: Mitigation of Silver Nanoparticle Toxicity to *Chlamydomonas reinhardtii* by Algal-Produced Organic Compounds

**DOI:** 10.1371/journal.pone.0074456

**Published:** 2013-09-23

**Authors:** Louise M. Stevenson, Helen Dickson, Tin Klanjscek, Arturo A. Keller, Edward McCauley, Roger M. Nisbet

**Affiliations:** 1 Department of Ecology, Evolution and Marine Biology, University of California Santa Barbara, Santa Barbara, California, United States of America; 2 Rudjer Boskovic Institute, Zagreb, Croatia; 3 Bren School of Environmental Science and Management, University of California Santa Barbara, Santa Barbara, California, United States of America; 4 Department of Biological Sciences, University of Calgary, Calgary, Alberta, Canada; University of California, Merced, United States of America

## Abstract

The vast majority of nanotoxicity studies measures the effect of exposure to a toxicant on an organism and ignores the potentially important effects of the organism on the toxicant. We investigated the effect of citrate-coated silver nanoparticles (AgNPs) on populations of the freshwater alga *Chlamydomonas reinhardtii* at different phases of batch culture growth and show that the AgNPs are most toxic to cultures in the early phases of growth. We offer strong evidence that reduced toxicity occurs because extracellular dissolved organic carbon (DOC) compounds *produced by the algal cells themselves* mitigate the toxicity of AgNPs. We analyzed this feedback with a dynamic model incorporating algal growth, nanoparticle dissolution, bioaccumulation of silver, DOC production and DOC-mediated inactivation of nanoparticles and ionic silver. Our findings demonstrate how the feedback between aquatic organisms and their environment may impact the toxicity and ecological effects of engineered nanoparticles.

## Introduction

Natural populations exert feedbacks on their environment through consumption, production and excretion. By modifying environments, organisms could significantly impact the fate and toxicity of nanomaterials. While direct impacts of toxicity have been widely assessed [1], understanding effects of environmental modifications on subsequent organismal responses to nanomaterials has been neglected.

Silver nanoparticles (AgNPs) comprise one of the fastest growing areas of nanotechnology [2] and are used in a broad range of consumer applications from home appliances to textiles, increasing their potential for environmental release. Studies have found considerable leaching of silver from consumer products containing AgNPs [3], [4]. These particles are utilized for their well-studied antimicrobial properties [5]–[7] through mechanisms such as cell wall damage [7], [8] and free radical production [9]. Studies have identified a toxic effect of AgNPs on marine and freshwater algal species[10]–[14], but these ignore the crucial feedback effect of algal species on the particles themselves.

We investigated the effect of citrate-coated AgNPs on the freshwater algae *Chlamydomonas reinhardtii* in different stages of growth in batch cultures. We found a nano-specific toxic effect of the AgNPs that cannot be explained by the presence of silver ions, a result that differs from past studies that have found that AgNP toxicity is mediated entirely through ionic silver (Ag^+^) [11], [12], [14]. Further, we found that extracellular molecules produced by the algal cells themselves mitigate both the nanoparticle-specific and ionic toxicity of AgNPs. This finding highlights how the feedback between freshwater organisms and their environment may impact the potential toxicity of AgNPs.

## Results and Discussion

### AgNPs are most Toxic to Earlier Stages of Algal Batch Culture Growth

**Figure 1:**
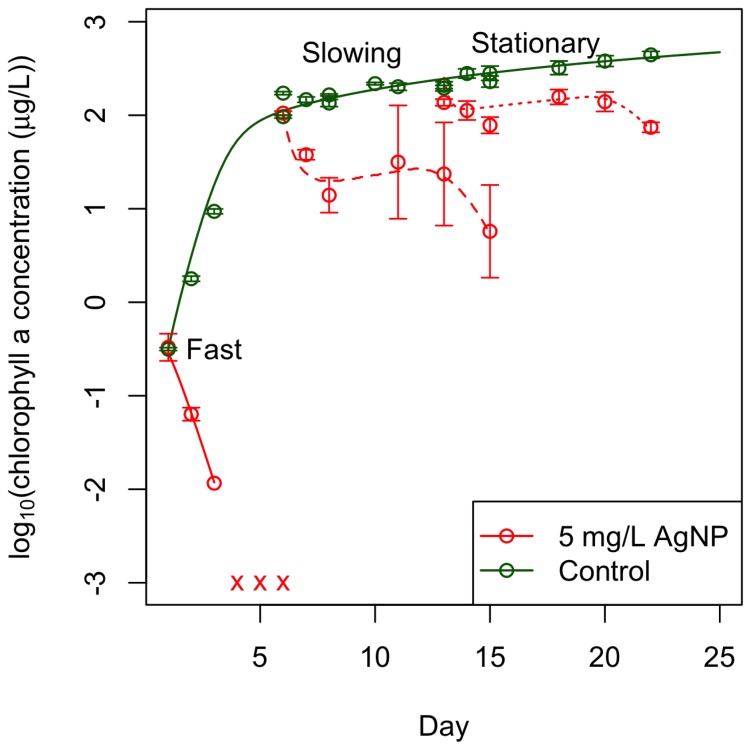
Citrate-AgNPs are more toxic to cultures in earlier stages of growth than in later stages and our dynamic model captures the algal dynamics. 5/L citrate-coated AgNPs were introduced to *C. reinhardii* batch cultures at three different stages of growth: fast growth (solid lines), slowing growth (large dashes), and stationary growth (small dashes). The dynamic model developed through analyses of these data captures the algal dynamics well with a single parameter set (lines). Batch cultures in slowing and stationary growth phases had grown for one and two weeks, respectively, prior to the start of the experiment and before the introduction of AgNPs. Time in this figure is represented as the absolute day of growth of the culture – all cultures were exposed on the same day of the experiment but on different days of growth (cultures in fast growth phase were dosed with AgNPs on day 1 of growth, cultures in slowing growth phase were dosed on day 6 of growth, and cultures in stationary growth phase were dosed on day 13 of growth). AgNPs caused complete mortality of cultures in fast growth phase within two days of introduction. For these cultures, chlorophyll measurements were below detectable limits (denoted by x) by day 3 but the culture was sampled through day 6. We measured concentrations of chlorophyll a to indicate algal cell viability and response to AgNPs because we empirically confirmed that chlorophyll a/cell ratios remain constant after day 5 of growth in algal cultures grown in the light and temperature environments used in this experiment. However, AgNPs had an initial toxic effect on cultures in slowing and stationary growth phases from which the cultures were able to recover until they declined again on days 8 and 10 of the experiment (days 13 and 15 of growth for cultures in slowing phase and days 20 and 22 of growth for cultures in stationary growth phase). The data points are averages from three replicate cultures and the error bars reflect their standard error.

We exposed algal batch cultures to 5 mg/L of 40 nm Citrate BioPure™ AgNPs (NanoComposix) during fast, slowing, and stationary phases of growth. The toxic response in an algal culture to 5 mg/L AgNPs depends on its growth stage. AgNPs were significantly more toxic to cultures in fast growth phase than cultures in later stages (Figure 1). We discovered that shaking algal cultures did not affect algal growth or response to AgNPs (Figure S1), so we continued our experimental analysis with unshaken cultures only. Cultures in slowing growth phase declined in population size over the first three days of exposure, partially recovered for a day, and then experienced a second decline. AgNPs caused a slight decline of cultures in stationary growth phase over the first three days of exposure, after which the cultures recovered for a day and then experienced a second decline similar to, but not as extreme as, cultures in slowing growth phase. Sondi & Salopek-Sondi (2004) found a similar effect of AgNPs on bacterial colonies, as AgNPs had greater bactericidal effects when the starting concentrations of colony-forming units was lower [7]. The response of algal batch cultures to an equimolar concentration of Ag^+^ in the form of AgNO_3_ was toxic to all cultures regardless of growth stage (Figure S2).

### Nano- or Ionic-specific Toxicity?

**Figure 2:**
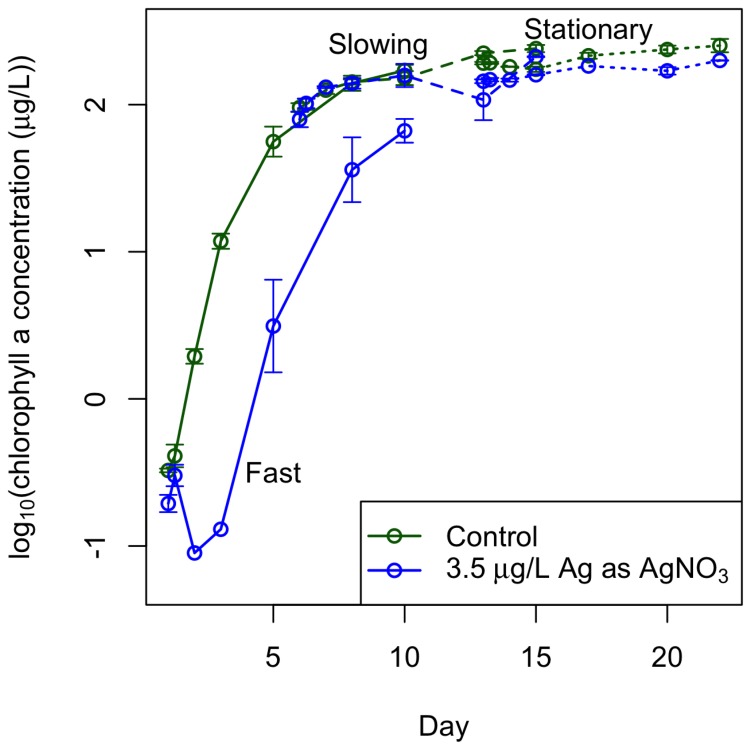
3.5 µg/L Ag^+^ has little to no effect on late stages of batch culture growth. 3.5 µg/L Ag^+^ in the form of AgNO_3_ was introduced to batch cultures in the same way as described in Figure 1. This concentration caused initial toxicity to cultures in fast growth phase, but these cultures were able to recover to the level of the control cultures in fast growth phase. This concentration had no visible effect on cultures in slowing and stationary growth phases. The data points are averages from three replicate cultures and the error bars reflect their standard error. The lines just connect the data points and help differentiate between culture in fast (solid), slowing (large dashes), and stationary (short dashes) growth phases.

While AgNP toxicity is well studied, a large question still remains as to whether the observed effect of AgNPs is due to some toxic mechanism of the particle itself (a nano-specific toxic effect) or due to the deleterious effects of Ag^+^ that can dissolve from AgNPs (an ionic toxic effect). The AgNP literature is divided on this question – some past studies have found that AgNP toxicity to marine and freshwater algae is mediated entirely through dissolution of silver ions from the particles [10], [12], [14] while others have found a nano-specific toxic effect of AgNPs on bacteria [5], [15] and on marine and freshwater algal species [10], [13]. In our experiment, introduced AgNPs were initially toxic to all stages of algal growth (Figure 1). To investigate whether this could be explained by the presence of Ag^+^ in the stock solutions of AgNPs that can be confused with a nanoparticle effect [16], we measured the Ag^+^ concentration in our stock AgNP solution and exposed algal batch cultures at the same three stages of growth to the measured Ag^+^ concentration in the form of AgNO_3_. The concentration, measured using Atomic Absorption Spectroscopy to be 3.5 µg/L Ag^+^, had little to no effect: later stages of growth experienced no decline, and cultures in fast growth phase only declined initially and were able to recover completely (Figure 2).

In addition to measuring the concentration of silver ions present in the stock solution of AgNPs, we measured dissolution of AgNPs in algal cultures in the three growth stages. Algal cells were removed to minimize loss of measurable Ag^+^ due to association with algal cells. The dissolved silver concentration was below 50 µg/L Ag^+^ for the first three days of introduction of the particles to the media (Figure S3); this slow dissolution is consistent with earlier studies of citrate-coated AgNP dissolution in environmentally-relevant media [17]. Since silver ions up to 100 µg/L do not have a significant toxic effect on cultures in late stages of growth (Figure S4), we conclude that Ag^+^ could not have caused the initial toxicity of all cultures. The initial decline is most likely due to a nano-specific effect of the AgNPs. A previous study on the effect of carbonate-coated AgNPs on the same freshwater algal species (*C. reinhardtii*) concluded that AgNP toxicity is mediated by Ag^+^ [12]. The authors also found, as we did, that the free Ag^+^ concentration could not account for the AgNP toxicity observed, but they characterized the AgNP toxicity as driven by Ag^+^ because the presence of cysteine, a strong Ag^+^ ligand, greatly reduced toxicity [12]. However, cysteine may be mitigating toxicity by binding to silver ions that have resorbed to the particle surface [18], a phenomenon that occurs with even citrate-coated particles [19]. This toxic mechanism would be considered a particle-specific effect in terms of DOC mitigation of Ag^+^ and AgNP toxicity incorporated in our model described later in this paper.

Previous researchers have expended a lot of effort to identify specific mechanisms of toxicity of AgNPs to microorganisms. Our study does not identify a specific toxic mechanism of AgNPs, however we did find that the nanoparticles themselves exert a toxic effect in addition to producing toxic silver ions. This finding corroborates other studies such as a different freshwater algal species found to accumulate AgNPs, and the particles exerted toxic effects intracellularly [10]. Intracellular uptake of AgNPs has been reported in bacteria [5], [15] and one study found limited uptake of AgNPs by *C. reinhardtii* [20]. Nanoparticle uptake may be greater for coated particles, like the citrate-coated AgNPs we used, due to an interaction between the polymer coating and the cell surface [21]. Intracellular accumulation of AgNPs may enhance dissolution [22] or facilitate damage by reactive oxygen species (ROS) produced by the nanoparticles [13].

### Algal-produced Dissolved Organic Carbon Mitigates the Toxicity of AgNPs

None of these mechanisms of toxicity recognize the environmental feedback of the organisms on the nanomaterials themselves, and the mechanisms alone cannot explain the differential toxicity we observed. Intracellular differences and/or differences in the external environment of algal cultures during the three investigated growth phases might explain the patterns of response to AgNP exposure. Intracellular differences could arise because late-stage cells are no longer absorbing limiting nutrients and are dividing slowly. They also experience a different extracellular environment because they have produced more organic products, especially dissolved organic carbon (DOC), produced by the algae during photosynthesis [23]. For example, we measured extracellular concentrations of dissolved organic carbon and found an increase from 8.94±0.004 mg-C/L in cultures on day 7 of growth to 22.5±0.003 mg-C/L in cultures on day 19 of growth (average of three measurements ± standard error).

**Figure 3:**
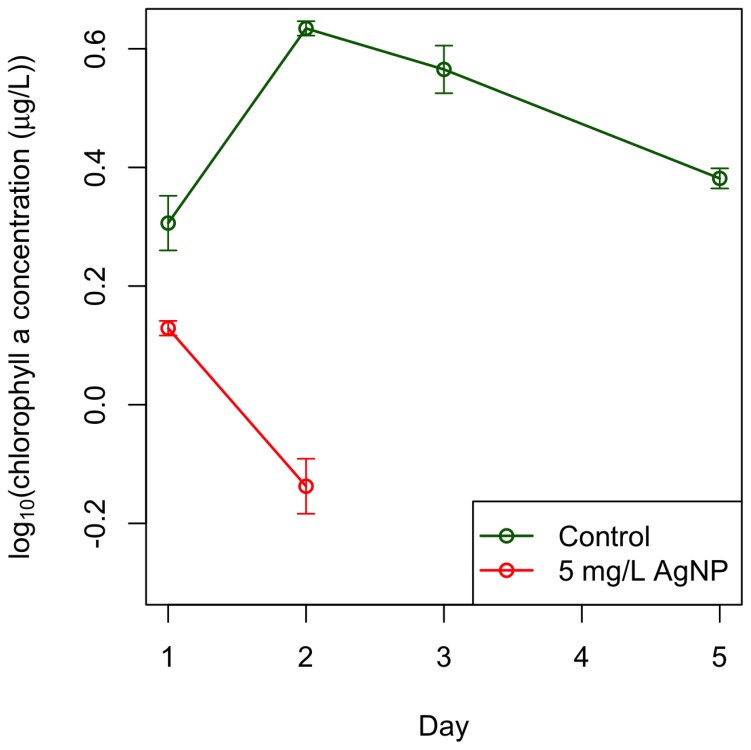
Removal of DOC from algal cultures in stationary growth restores the toxicity of AgNPs. AgNPs cause complete mortality of cells in stationary growth (red) after removal of organic material and resuspension of algal cells in media without nutrients. Control cultures (green), which were also centrifuged and resuspended in media without nutrients, persisted for at least 5 days. This pattern of toxicity is very similar to the rate of decline of cultures in fast growth phase exposed to 5 mg/L AgNP (Figure 1). This finding indicates that the difference in toxicity observed between growth stages of the algae is most likely due to differences in the extracellular environments of the growth stages. The data points are averages from three replicate cultures and the error bars reflect their standard error.

We favor the hypothesis that extracellular differences are the primary cause of the differential toxicity observed using exposure experiments that manipulated the DOC concentration of the environment. We centrifuged a culture in stationary growth phase, removed the supernatant containing organic material, and re-suspended the algal cells in synthetic freshwater media without nitrogen or phosphorus to reduce nutrient uptake. We then exposed the algal cells in this “new” stationary growth phase to 5 mg/L AgNPs and found that the algal cells died within two days (Figure 3) – the same toxicity pattern as cultures in fast growth phase seen previously (Figure 1). Control cultures, which had also been centrifuged and re-suspended, persisted for at least 5 days (Figure 3). The differential response in initial toxicity between the stages of algal growth is explained primarily by differences in the extracellular environment, such as extracellular DOC.

DOC can mitigate the toxicity of AgNPs directly or indirectly. DOC could be promoting the formation of less-toxic aggregates [7] – AgNPs remained as single, unassociated particles in cultures in fast growth phase but aggregated in cultures in slowing and stationary growth (Figure S5). DOC has also been shown to physically interact with nanoparticles [5] and complex with Ag^+^ [24], decreasing their toxicity [11], [25], or by interrupting the mode of toxicity of both forms of silver. DOC could prevent a toxic effect on the algal cells by limiting particle-cell interactions [5], [26] or uptake, or by acting as a sink for ROS [27]. Humic acids decreased the toxicity of AgNPs to *Oryzias latipes* embryos by coating the surfaces of the AgNPs and forming bridges between particles; an interaction that may disrupt the release of Ag^+^ from the particles or prevent the AgNPs from penetrating the embryos [28]. Algal-produced expolymeric substances from a marine diatom mitigated the toxic effect of Ag^+^, and the natural organic compounds used to complex with Ag^+^ may have actually coated the AgNPs themselves, protecting the diatom from AgNPs [11].

### Dynamic Model of Feedback

**Figure 4:**
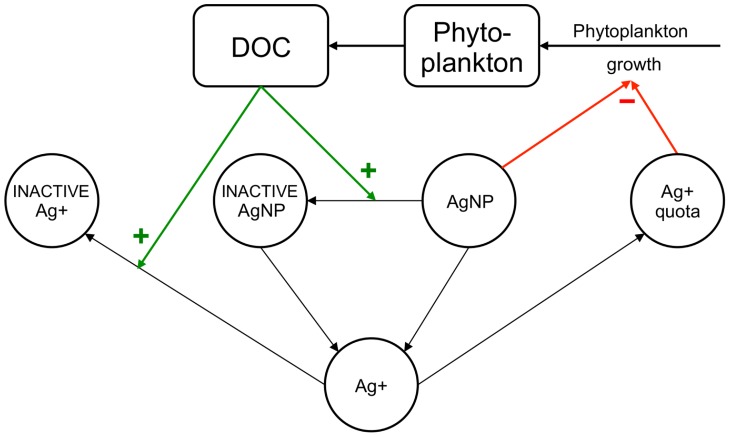
Schematic of dynamic model of environmental feedback. Phytoplankton grow and produce DOC, which inactivates AgNPs and silver ions (Ag^+^). Both active and inactive AgNPs dissolve, introducing Ag^+^ into the environment. Environmental Ag^+^ is either made inaccessible to phytoplankton (inactivated Ag^+^) or bioaccumulated by the phytoplankton (entering the Ag^+^ quota). The bioaccumulated Ag^+^ and the still active AgNPs exert toxic effects on the phytoplankton.

**Figure 5:**
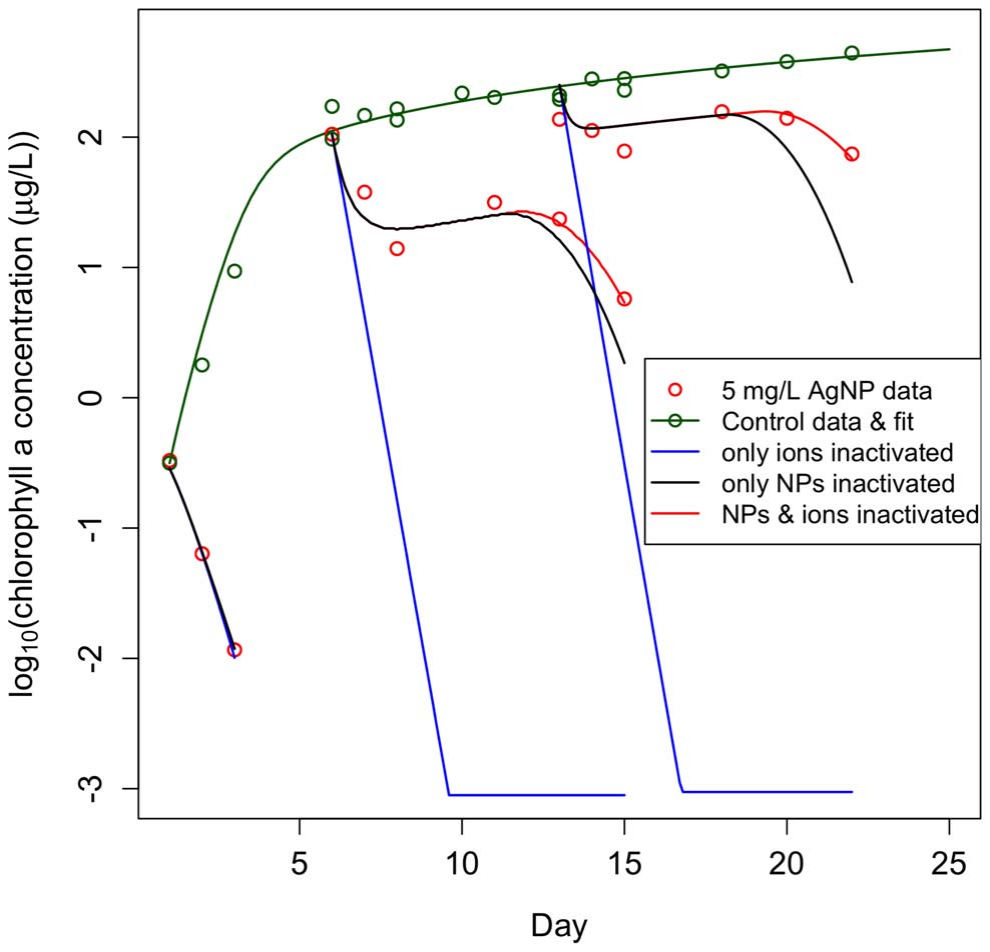
Model predictions with inactivation mechanisms of DOC on AgNP and Ag^+^ separately *and* in unison. Model simulations demonstrate the significance of DOC inactivation of AgNPs *and* Ag^+^ (red lines). The simulations suggest that DOC mitigation of nanotoxicity provides a much stronger feedback than mitigation of ionic toxicity: while the model without ionic mitigation (black lines) generally follows the observations and only predicts the second dip slightly sooner, the model without AgNP inactivation (blue lines) radically departs from the observations, with the population going extinct by day three of the exposure.

We developed a dynamic, process-oriented model that demonstrates how the processes identified through our experiments and the feedbacks shown in Figure 4 could lead to the observed patterns in phytoplankton growth (Figure 1), in particular the “double dip” in algal density that followed exposure in the later stages of batch culture. The model includes phytoplankton growth, DOC production, toxicity and dissolution of nanoparticles, bioaccumulation and the associated toxicity, and feedback on toxicity through two mechanisms: inactivation of ionic- and nano-silver by the phytoplankton-produced DOC. Toxicity is characterized as additional mortality with contributions from exposure to both bioaccumulated (ionic) and nano-silver. We used the model to illustrate the effects of the two inactivation mechanisms acting in concert and separately (Figure 5). Further details, model equations, fitting methodology, and parameter values are in Section 6 in Text S1 and Tables 1 and 2.

**Table 1:** Simple dynamic model describing the feedbacks.

**State variables**	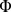	Chlorophyll-a in algal population (µg-Chl-a L^−1^)
	D	Dissolved organic carbon (DOC) concentration (mg-C L^−1^)
	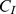	Concentration of dissolved silver (mg-Ag L^−1^)
	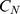	Concentration of bioavailable AgNPs (mg-Ag L^−1^)
	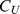	Concentration of inactivated AgNPs (mg-Ag L^−1^)
	q	“quota” of bioaccumulated Ag in algae (mg-Ag (µg-Chl-a)^−1^)
**Rates**	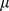	exposure-related specific mortality rate of algae (day^−1^)
	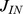	rate of dissolution of AgNPs (mg-Ag L^−1^ day^−1^)
	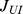	rate of inactivation of ionic silver (mg-Ag L^−1^ day^−1^)
	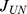	rate of inactivation of AgNPs (mg-Ag L^−1^ day^−1^)
	P	rate of production of DOC by algae (mg-C day^−1^)
**Balance Equations**	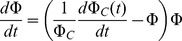	Algae
	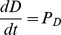	Dissolved organic carbon (DOC)
	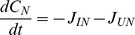	Bioavailable AgNPs
	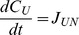	Inactivated AgNPs
	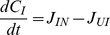	Ionic silver
	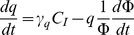	Quota
**Model functions**	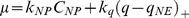	Exposure-induced mortality
	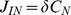	AgNP dissolution
	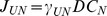	AgNP inactivation by DOC
	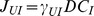	Inactivation of ionic silver
	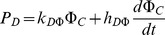	DOC production
	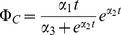	Chl-a in control population

Subscripts used are N:silver nanoparticles; I: ionic silver; U: non-bioavailable (inactivated) silver. In the balance equations, 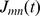
denotes the rate of transformation of silver in state *n* to state *m*. Parameters are defined in Table 2. Note: the notation [*x*]_+_ means use the value of x if it is positive, otherwise set to zero.

**Table 2:** Initial conditions and parameter values for the model fits shown in Figures 1 and 5.

symbol	name	value
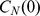	Initial total silver	4.9965 mg-Ag L^−1^
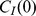	Initial ionic silver	0.0035 mg-Ag L^−1^
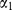	Parameter in phytoplankton production rate	19.34 µg-Chl-a day^−1^
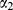	Parameter in phytoplankton production rate	1.287 day^−1^
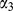	Parameter in phytoplankton production rate	240.9
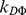	Parameter in DOC production rate	0.0837 mg-C (µg-Chl-a)^−1^ day^−1^
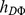	Parameter in DOC production rate	9.77  10^−4^ mg-C (µg-Chl-a)^−1^
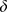	AgNP dissolution rate	0.0091 day^−1^
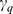	Silver bioaccumulation rate	1.56  10^−4^ L (µg-Chl-a)^−1^ day^−1^
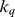	Toxicity parameter for bioaccumulated silver	1.14  10^4^ (µg-Chl-a) (mg-Ag)^−1^ day^−1^
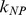	AgNP toxicity parameter	0.692 L (mg-Ag)^−1^ day^−1^
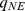	No-effect quota	8  10^−5^ (mg-Ag) (µg-Chl-a)^−1^
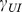	Silver ion inactivation rate	0.0081 day^−1^
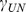	AgNP inactivation rate	0.1788 day^−1^

Parameters 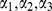 were estimated from the growth curves of the control cultures. Parameters 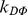 and 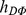 were calculated from measured DOC values. Other parameters were minimizing the residual sum of squares between model output and chlorophyll a data for all three treatments simultaneously.

The model makes the following assumptions on processes, with the formulae implementing them detailed in the “Model Functions” section of Table 1:

#### Phytoplankton growth

Chlorophyll a (Chl-a) is used as a surrogate for phytoplankton population size or biomass. The growth curve of the control population represents the combined effects of primary production and natural mortality; we use an empirical fit to this curve as the baseline for phytoplankton dynamics and then model additional mortality due to toxicity.

#### DOC excretion

The rate of excretion of DOC from cells to the environment depends on the rates of photosynthesis, maintenance, and growth. In the absence of time resolved empirical data, we assume that the rate of DOC production can be described as a sum of a terms proportional to population size and growth rate.

#### Dissolution of AgNPs and Bioaccumulation of dissolved silver

Both processes are described by first order kinetics.

#### Toxicity of silver

Toxic effects are represented as additional mortality terms proportional to the concentration of AgNPs in the environment and to bioaccumulated ionic silver above a minimum no-effect “quota”.

#### Inactivation of nanoparticles and ions

DOC affects toxicity by reducing effective exposure. The inactivation rates are proportional to DOC concentration.

The model has a minimal representation of chemical processes, yet captures the dynamics remarkably well with a single parameter set (Figure 1) and enables us to distinguish effects of nano- and ionic toxicity (Figure 5). Seven parameters (dissolution of NPs (δ), deactivation rates of NPs and ions 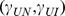, nano-particle and ionic toxicities 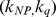, no-effect quota (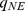), and bioaccumulation rate (*γ_q_*)) were estimated by minimizing the residual sum of squares between model output and Chl-a data for all three treatments simultaneously (see Table 2 for parameter values). The model captures the essentials of the dynamics of the system: phytoplankton populations recover if high DOC levels are present, and then - after a few days - decline again, with the slope of the decline smaller for higher DOC levels. It is sufficiently simple to be coupled as a module to existing nutrient-phytoplankton-zooplankton models [29], and thus to contribute to predicting effects of nanoparticle exposure in more complex food webs.

## Conclusions

Through empirical and quantitative analyses, we found that AgNPs are more toxic to algal batch cultures in earlier stages than later stages of growth due to the protective effect of algal-produced DOC. It is worth noting that even though the concentration of AgNPs we used (5 mg/L) is high compared to predicted environmental concentrations [3], [4], we expect the mitigating effect of algal-produced DOC to operate at lower concentrations and lessen the toxicity of AgNPs to other freshwater organisms that can be more susceptible, such as zooplankton [30]. One study identified a mitigating effect of DOC on AgNP toxicity to *Daphnia* [31], and we found a qualitatively consistent protective effect of algal-produced DOC on the toxic effect of AgNPs to *Daphnia pulicaria* in preliminary studies (unpublished data). Algal productivity driving AgNP toxicity is particularly important considering the natural cycling of algal and zooplankton populations [32]. Our work emphasizes the importance of the effect of the focal organism on the toxicant and highlights the need for experiments exposing multiple species from the same environment to nanoparticles, since byproducts of one species may influence nanoparticle toxicity on all organisms in that environment.

## Materials and Methods

### 1. Batch Culture Setups and Treatment Groups

Batch cultures of *Chlamydomonas reinhardtii* were grown in 500 mL Erlenmeyer flasks. New cultures were inoculated with a cell concentration of 10^6^ cells/L. Cultures used for inoculation were counted using a hemocytometer and then diluted into 250 mL of fresh COMBO media [33] to 10^6^ cells/L. One and two weeks prior to the start of the experiment, new batch cultures were started and grown undisturbed in the experimental setup used during the experiment itself. To distinguish the effect of AgNPs on different stages of algal growth, the experiment began when these cultures were one and two weeks old. New cultures were inoculated with algal cells the day the experiment started. New cultures were in fast growth phase, one week old cultures were in slowing growth phase, and two week old cultures were in stationary growth phase.

### 2. Measurement of Chlorophyll a Concentrations

We measured concentrations of chlorophyll a with a Gemini XPS Fluorescence Microplate Reader (Molecular Devices). We measured the fluorescence of four 200 µL samples of each culture, averaged these values, and converted to concentrations of chlorophyll a (µg/L) using a standard curve calibrated for our instrument with Turner Designs Liquid Primary Chlorophyll A Standards.

### 3. Dissolved Organic Carbon Removal Experiment

To remove DOC, we centrifuged samples of a two-week-old algal batch culture on 7,000 rpm for 8 minutes on an Eppendorf 5430R Centrifuge two times, pouring off the supernatant and resuspending the pellet in COMBO media without nitrogen or phosphorus after each spin. We spun the samples twice because we found that a large concentration of DOC was removed after two sequential spins, while a third spin removed a negligible amount of DOC (unpublished data). We then diluted this sample to 10^7^ cells/L in media without nitrogen and phosphorus. We decreased cell concentration because pilot experiments with this protocol showed that the initial, higher algal cell concentrations (10^9^ cells/L) rapidly produced a significant amount of DOC that caused AgNPs to aggregate within a day. Control and 5 mg/L AgNPs exposed cultures were sampled using the same experimental setup described in Section 1 of Text S1. These late-stage cells were exposed to AgNPs that did not aggregate (mean particle size remained around 40 nm; see Section 5 in Text S1 for AgNP size measurement protocol).

### 4. Measurements of the Dissolution of Silver Ions from the AgNPs

To avoid underestimating the dissolution of free silver ions by missing Ag^+^ absorbed by the algal cells themselves, we filtered all of the algal cells out of new (fast growth phase), one week old (slowing growth phase) and two week old (stationary growth phase) cultures using 5 micron filters (Millipore MF-Mixed Cellulose Ester Membrane filters). We added 5 mg/L of 40 nm citrate-coated AgNPs and took samples at the same frequency we sampled the initial AgNP experiment using the same batch culture sampling apparatus described previously (see Section 1 in Text S1). We removed 15 mL of the culture for every sample and spun these samples down in acid-washed Amicon centrifugal filter units (Amicon Ultra-15, 10,000 NMWL) for 30 minutes at 5,550 rpm. We added 0.1% nitric acid to the sample and stored it in the dark until digestion. For the digestion process, we added 3 parts HNO_3_ and 1 part HCl to every sample and heated the samples in a Hach Reactor (DRB 200 Reactor) for 30 minutes at 85°F. We then measured the final volume. These samples were analyzed by the Marine Science Analytical Lab at UCSB using Atomic Absorption Spectrophotometers with graphite furnace atomization (Varian Instruments AA240Z).

### 5. Dynamic Model

The model simulations and the parameter estimation were performed using proprietary code written in MATLAB.

## Supporting Information

Figure S1 **Shaking algal cultures has no effect on control or AgNP cultures.** There was no difference between AgNP (a) and control (b) cultures on shaker tables (red) and kept stationary (green). The data points are averages from three replicate cultures and the error bars reflect their standard error.
(TIF)

Figure S2 **An equimolar to 5 mg/L silver concentration of Ag^+^ was toxic to all algal batch cultures.** An equimolar concentration of Ag^+^ in the form of AgNO_3_ was toxic to algal cultures in all growth stages (blue). Cultures in fast growth phase never registered a positive chlorophyll reading so the AgNO_3_ treatment is not represented on this graph. We also exposed cultures to an equimolar concentration of NO_3_ as the AgNO_3_ treatments to compare to control for this addition of nitrogen (green), which algal cells can use for growth. We did not see a difference between control cultures and cultures with an NO_3_ addition. The data points are averages from three replicate cultures and the error bars reflect their standard error.
(TIF)

Figure S3 **Concentration of dissolved silver from AgNPs introduced to algal cultures with algal cells removed.** We removed the algal cells from cultures in fast, slowing, and stationary growth phases in order to minimize loss of Ag^+^ in our measurement due to association with algal cells.
(TIF)

Figure S4 **The effect of 10 (a), 50 (b), and 100 (c) µg/L Ag^+^ on algal cultures.** These concentrations of Ag^+^ were introduced to batch cultures in the form of AgNO_3_ (blue) in the same way described in Section 1 of Text S1. All three concentrations caused complete mortality of cultures growing in fast growth phase within two days of introduction (chlorophyll measurements were below detectable limits, denoted by x, on day 3 of new cultures exposed to 100 µg/L AgNO_3_). However, 10 and 50 µg/L Ag^+^ in the form of AgNO_3_ had negligible effect on cultures growing in slowing and stationary growth phases (a and b). 100 µg/L Ag^+^ in the form of AgNO_3_ had no effect on cultures in stationary growth phase but was initially toxic to cultures growing in slowing growth phase, however the cultures were able to partially recover (c). The data points are averages from three replicate cultures and the error bars reflect their standard error.
(TIF)

Figure S5 **AgNPs remained as single particles in cultures in fast growth phase and aggregated in later stages.** The 40 nm particles remained unassociated in cultures in earlier stages of growth but aggregated up to 130 nm in later stages of growth. The data points are averages from three replicate samples and the error bars reflect their standard error.
(TIF)

Text S1 (DOCX)
