# Supplementary material for: Environmental Feedbacks and Engineered Nanoparticles: Mitigation of Silver Nanoparticle Toxicity to Chlamydomonas reinhardtii by Algal-Produced Organic Compounds
Source: PLoS One. 2013 Sep 23;8(9):e74456. doi: 10.1371/journal.pone.0074456 (PMC3781102; doi:10.1371/journal.pone.0074456)
Supplement: Text S1 — (DOCX) [file pone.0074456.s006.docx]

The overall description of our algal batch culture experiments is presented in the Methods section. In Sections 1-5 of the SI we describe the sampling and experimental analyses. In Section 6 we detail potential expansions of the dynamic model that demonstrates the feedback mechanism.

**1. Batch culture setups for all algal batch culture experiments**

Samples were taken directly from the algal batch cultures every day for the first 3 days, and on a Monday-Wednesday-Friday schedule for the rest of the experiments. The sampling setup allowed us to sample directly from the flasks without opening them, thus minimizing contamination. The setup consists of a 9-inch glass pipette held in place by a foam stopper at the top of the 500 mL Erlenmeyer flask with Tygon tubing attached to the end of the pipette. We secured two plastic 10 mL syringes (Exel International Luer Lock syringe) at the other end of the tube with a polypropylene T-valve (Thermo Scientific Nalgene Tubing T-type connectors). By sucking air out with one syringe, we were able to extract a sample with the other plastic syringe. We swirled cultures prior to sampling them to suspend algal cells and ensure that we were taking a homogenous sample. The whole setup was autoclaved prior to the experiment to ensure sterility.

Batch cultures of *C. reinhardtii* were grown under constant light provided by fluorescent “growing” lights (Philips T8 Natural Light 32 W fluorescent lamps) suspended above the cultures on wooden sawhorses. We measured photosynthetically active radiation (PAR) levels across the entire experimental setup using a digital light meter (Extech Instruments Model 401025) to ensure that each flask grew in approximately equivalent PAR levels. PAR readings across the experiment varied by approximately 10% Lux.

**2. The effect of 5 mg/L AgNP on three stages of batch culture growth**

Nanoparticles in stationary cultures could sink to the bottom and not homogenously interact with algal cells. To examine the effect of shaking on particle behavior and algal toxicity, we placed half of the cultures on shaker tables (Thermo Scientific MaxQ 3000 orbital shaker) for the duration of the experiment and the other half on stationary platforms of the same height as the shaker tables. We did not find an effect of shaking the cultures on control or AgNP-treated cultures (Figure S1).

The first algal batch culture experiment involved four treatment groups: 1) citrate-coated AgNPs, 2) an equimolar concentration of silver nitrate (AgNO_3_), 3) an equimolar concentration of nitrate (NO_3_) to the AgNO_3_ treatment, and 4) control. Treatment 1 tested the effect of 5 mg/L citrate-coated silver nanoparticles (40 nm Citrate BioPure**™** Silver from NanoComposix, Inc). The aim of treatment 2 was to investigate a “nano” versus “silver” effect of these particles; we therefore dosed the algal cultures with an equimolar silver concentration to the 5 mg/L concentration of AgNPs in the form of AgNO_3_. Treatment 3 controlled for possible effects of the addition of nitrate in treatment 2 that could affect algal growth; we exposed a set of algal cultures to an equimolar concentration of NO_3_ to the AgNO_3_ treatment.

The equimolar to 5 mg/L silver concentration of Ag^+^ in the form of AgNO_3_ was toxic to all algal batch cultures regardless of growth stage and we did not see a difference between control cultures and cultures with an NO_3_ addition (Figure S2).

**3. Measurements of the dissolution of silver ions from the AgNPs**

The concentration of Ag^+^ in our 40 nm citrate-coated AgNP stock was measured by first separating the Ag^+^ from the AgNPs through ultracentrifugation and then measuring the concentration of this separated ionic fraction on an Atomic Absorbance Spectrophotometer.

In order to estimate the total dissolution of silver ions from the AgNPs throughout the duration of the experiment, we conducted a follow up experiment to measure the dissolution of Ag^+^ from the nanoparticles in the organic environment of the three stages of batch culture growth. Please refer to the Materials & Methods section in the main text of this study for details on the experimental setup and sample collection and processing. Dissolution of the AgNPs in the organic environment of algal batch cultures was relatively slow (Figure S3), with a maximum Ag^+^ concentration of 90 μg/L after 10 days of dissolution in the organic material from a culture growing in stationary growth phase. We also found an interesting pattern in which the concentration of dissolved ions actually decreased initially for AgNPs in fast growth phase and cultures in stationary growth phase. Numerous other studies of AgNP dissolution have also observed this decrease in the dissolved ionic fraction and it is thought to be due to either released Ag^+^ rejoining existing AgNPs [[1-3](#_ENREF_1)] and/or complexation of Ag^+^ with various ligands, including chloride and DOC [[4](#_ENREF_4)], which may or may not have been removed by the Amicon filter. However, measurements from algal cultures in slowing growth phase do not reflect this pattern for which we do not have an explanation.

**4. The effect of 10, 50 and 100 μg/L Ag^+^ in the form of AgNO_3_**

New, one week, and two week old cultures were inoculated for growth prior to the experiment in the same manner as described in Materials & Methods. The same batch culture sampling setup was employed and cultures were sampled for chlorophyll a measurements at the same frequency as previously described (see Supplementary Section 1). We looked at the effect of 3.5 (Figure 2), 10, 50, and 100 μg/L Ag^+^ in the form of AgNO_3_ (Figure S4). We found little to no effect of 3.5 μg/L Ag^+^ (Figure 2). All other concentrations tested were toxic to cultures in fast growth phase, but only 100 μg/L Ag^+^ had an effect on later stages of batch culture growth (Figure S4).

**5. Measurement of AgNP size**

We conducted an experiment to measure the size of the AgNPs in algal batch culture using a higher concentration of AgNPs than was used in our original experiment, since samples of the 5 mg/L concentration used for our original experiment were not of a high enough concentration for the Malvern Instruments Zetasizer Nano ZS90 to read accurately (the small attenuator value meant we could not have confidence in these measurements). We grew batch cultures of *C. reinhardtii* for one, two, and three weeks prior to this follow-up experiment. We employed two different filter techniques to estimate if filtration (necessary to remove algal cells prior to nanoparticle size measurements) removed large aggregates of nanoparticles. AgNPs in samples from which algal cells had already been removed still experienced the level of organic material produced by an algal culture. On the day of the experiment, we drew two 1.5 mL samples of new (fast growth phase), one week old (slowing growth phase), two week old (stationary growth phase), and three week old (late stationary growth phase) cultures. We then added 20 mg/L AgNPs to half of these samples, which were left for an hour under fluorescent lights and then filtered. For the other half, we filtered algal cells out of these samples using a 5 micron filter (Millipore MF-Mixed Cellulose Ester Membrane filters) prior to the addition of 20 mg/L AgNPs. We left all of these samples under fluorescent growing lights for an hour, filtered the half of the samples that still contained algal cells, and ran all of the samples on the zetasizer. We did not find a significant difference between samples that were filtered before AgNP addition and those filtered after AgNP addition (unpublished data). We found that the particles aggregated in later stages of algal batch culture growth, with a maximum size around 130 nm (Figure S5; data from algal culture that were filtered after AgNP addition).

**6. Dynamic model of feedback**

The model is deliberately simplistic, as its aim was to find a minimal suite of mechanisms consistent with our observations. Potentially important elaborations and their likely consequences include:

• variable strength and types of silver ion complexation by the DOC (affects effective exposure),

• dependence of NP dissolution rate on DOC (faster dissolution would lead to faster bioaccumulation),

• DOC introduced to the environment as the result of phytoplankton mortality (additional DOC would accelerate inactivation of both AgNPs and ions),

• a possible no-effect concentration for nano-particle exposure (transition between the initial population decline and recovery phases would be more abrupt),

• bioaccumulation of AgNPs into the algal cells themselves where they could exert toxic effects intracellularly

**References:**

1. Lee YJ, Kim J, Oh J, Bae S, Lee S, et al. (2012) Ion-release kinetics and ecotoxicity effects of silver nanoparticles. Environ Toxicol Chem 31: 155-159.

2. Li X, Lenhart JJ (2012) Aggregation and dissolution of silver nanoparticles in natural surface water. Environ Sci Technol 46: 5378-5386.

3. Liu J, Hurt RH (2010) Ion release kinetics and particle persistence in aqueous nano-silver colloids. Environ Sci Technol 44: 2169-2175.

4. Janes N, Playle RC (1995) Modeling silver binding to gills of Rainbow trout (Oncorhynchus mykiss). Environmental toxicology and chemistry.

**Supporting information legends:**

**Figure S1. Shaking algal cultures has no effect on control or AgNP cultures.** There was no difference between AgNP (a) and control (b) cultures on shaker tables (red) and kept stationary (green). The data points are averages from three replicate cultures and the error bars reflect their standard error.

**Figure S2. An equimolar to 5 mg/L silver concentration of Ag^+^ was toxic to all algal batch cultures.** An equimolar concentration of Ag^+^ in the form of AgNO_3_ was toxic to algal cultures in all growth stages (blue). Cultures in fast growth phase never registered a positive chlorophyll reading so the AgNO_3_ treatment is not represented on this graph. We also exposed cultures to an equimolar concentration of NO_3_ as the AgNO_3_ treatments to compare to control for this addition of nitrogen (green), which algal cells can use for growth. We did not see a difference between control cultures and cultures with an NO_3_ addition. The data points are averages from three replicate cultures and the error bars reflect their standard error.

**Figure S3. Concentration of dissolved silver from AgNPs introduced to algal cultures with algal cells removed.** We removed the algal cells from cultures in fast, slowing, and stationary growth phases in order to minimize loss of Ag^+^ in our measurement due to association with algal cells.

**Figure S4. The effect of 10 (a), 50 (b), and 100 (c) μg/L Ag^+^ on algal cultures.** These concentrations of Ag^+^ were introduced to batch cultures in the form of AgNO_3_ (blue) in the same way described in Supplementary Section 1. All three concentrations caused complete mortality of cultures growing in fast growth phase within two days of introduction (chlorophyll measurements were below detectable limits, denoted by x, on day 3 of new cultures exposed to 100 μg/L AgNO_3_). However, 10 and 50 μg/L Ag^+^ in the form of AgNO_3_ had negligible effect on cultures growing in slowing and stationary growth phases (a and b). 100 μg/L Ag^+^ in the form of AgNO_3_ had no effect on cultures in stationary growth phase but was initially toxic to cultures growing in slowing growth phase, however the cultures were able to partially recover (c). The data points are averages from three replicate cultures and the error bars reflect their standard error.

**Figure S5. AgNPs remained as single particles in cultures in fast growth phase and aggregated in later stages.** The 40 nm particles remained unassociated in cultures in earlier stages of growth but aggregated up to 130 nm in later stages of growth. The data points are averages from three replicate samples and the error bars reflect their standard error.
